# Clear Cell Papillary Renal Cell Carcinoma in the Bilateral Native Kidneys after 2 Years of Renal Transplantation: Report of a Case and Review of the Literature

**DOI:** 10.1155/2011/387645

**Published:** 2011-06-30

**Authors:** Zhanyong Bing, John E. Tomaszewski

**Affiliations:** Department of Pathology and Laboratory Medicine, Hospital of the University of Pennsylvania, 3400 Spruce Street, Philadelphia, PA 19104, USA

## Abstract

Renal transplantation increases the probability of malignant tumors by about 2–4-fold overall with a much higher rate for renal epithelial malignancy. Renal tumors in renal transplant recipients are commonly conventional clear cell or papillary renal cell carcinoma. Clear cell papillary renal cell carcinoma is a recently described unique renal epithelial neoplasm with scant eosinophilic or moderate amount of clear cytoplasm and pyknotic small nuclei oriented commonly toward the apical surfaces. No such tumor has been reported in renal transplant recipients. In this paper, we describe a clear cell papillary renal cell carcinoma involving bilateral native kidneys in a patient who had received a renal transplant 2 years earlier. Clear cell papillary renal cell carcinoma commonly presents with low pathologic stage and lower Fuhrman grade and is clinically indolent. Additional cases are needed to evaluate the clinical behavior of this type of tumor in renal transplant recipients.

## 1. Introduction

Renal transplantation has greatly improved the lives of the patients with end-stage renal disease; however, their life expectancies are still not at par with general population. One of factors that contribute to such outcome is increased tendency to develop malignancy. The incidence of cancer development in renal transplants is increased 2–4-fold [1–3] overall, while there is greater increase in the incidence of kidney malignancies (15-fold) in renal transplants. 

 Clear cell papillary renal cell carcinoma (CCPRCC) is a recently described low-grade renal cell carcinoma [4–9]. It is usually strongly positive for cytokeratin 7 (CK7) and mostly negative for CD10. Genetically no deletion of chromosome 3p or loss of chromosome Y has been found [5]. In addition, Aydin et al. studied gain of chromosome 7 or 17 in such tumors and found only low copy number of such chromosomal changes in 1 out of 10 cases [4]. This unique type of tumor has been described in kidneys with [9] or without [5] end-stage renal diseases. Such type of tumor has not been reported in the patients with renal transplants. In this paper, we described a renal transplant recipient with multiple cystic clear cell papillary renal cell carcinomas in both native kidneys.

## 2. Clinical History

The patient was a 67-year-old lady with end-stage renal disease secondary to type II diabetes and hypertension. She received a cadaveric kidney from a 51-year-old man who died from an intracranial event. The patient was immunosuppressed with tacrolimus/mycophenolate/prednisone. Tacrolimus levels were monitored for toxicity. Two years later, the patient was found to have bilateral renal masses and underwent bilateral nephrectomies of the native kidneys. The patient was alive without recurrence of carcinoma 21 months status after surgery. 

### 2.1. Pathology Findings

#### 2.1.1. Macroscopic Findings

The left kidney measured 8.0 × 3.2 × 3.3 cm. The kidney was atrophic. The cortical thickness was 2 mm. There were 4 cystic lesions in the kidney which measured 0.6 cm, 0.5 cm, 0.4 cm, and 0.3 cm in diameter. 

The right kidney measured 8.6 × 5.0 × 3.0 cm. The kidney was also atrophic. There was an exophytic polar mass which measured 2.2 × 1.3 × 1.0 cm, and the mass was partially cystic and partially gelatinous with a well-defined tan-white capsule. Adjacent to this mass, there was a cystic lesion measuring 0.9 cm in diameter. At the opposite pole, there was a cystic lesion measured 1.1 cm in diameter and multiple cysts ranging from 0.3 cm to 0.5 cm in diameter. In addition, in the middle pole, there was a gelatinous lesion measuring 0.3 cm in diameter.

#### 2.1.2. Microscopic Findings

**Figure 1:**
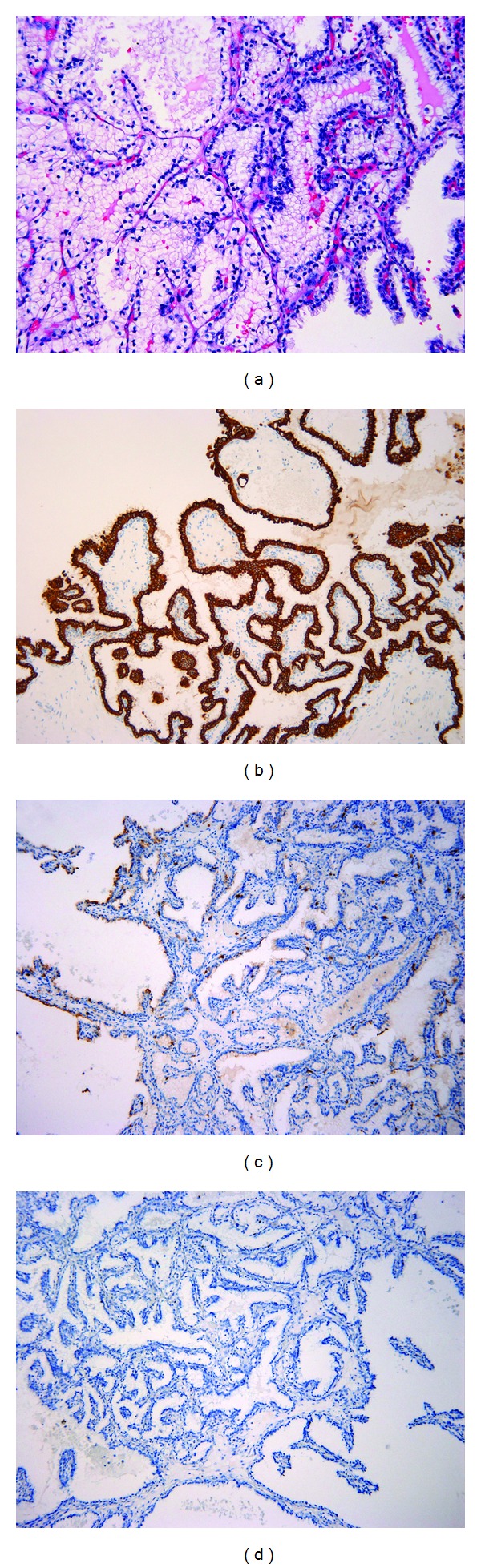
Clear cell papillary renal cell carcinoma in native kidneys of a renal transplant recipient. (a) Single layers of clear cells with moderate amount of clear cytoplasm and pyknotic nuclei oriented toward the apical surfaces, H&E 400×; (b) diffuse CK7 positivity, 100×; (c) weak CD10 stain, 100×; (d) negative RCC stain, 100×.

There were four cystic lesions in each kidney. In the left kidney, one tumor had a complete capsule. The remaining tumors did not have any capsules. In the right kidney, two tumors were completely encapsulated while one had a partial capsule and one no capsule. Some cysts contained colloid-like secretion. The tumors did not have a prominent fibrotic stroma. The tumors showed several morphologic patterns including tubules, acini, papillae, or ribbons lined with single layer of clear cells. The clear cells showed scant eosinophilic or moderate amount of clear cytoplasm. The nuclei of the clear cells were pyknotic, small, and mostly oriented toward the apical surfaces (Fuhrman grade 1). No necrosis or sarcomatoid changes were identified. No microscopic vascular invasion or perinephric or renal sinus invasion was seen. All of resection margins were negative. Immunohistochemically, the tumor cells showed diffuse positivity for CK7 and E-cadherin, weakly positive for CD10, and negative staining for renal cell carcinoma antigen (RCC). The background kidneys showed end-stage changes including extensive global glomerulosclerosis, tubular atrophy, and diffuse interstitial fibrosis (see Figure 1).

## 3. Discussion

Clear cell papillary renal cell carcinoma (CCPRCC) is a recently described low-grade renal cell cancer [4, 5, 7–9]. It can occur in both end-staged kidneys [9] and normal kidneys [5]. No coagulative necrosis, sarcomatoid changes, or vascular invasion has been identified [4]. 

The tumors are commonly cystic, with multiple growth patterns, including cysts, tubules, acini, papillae, and clear cell nests [4]. Branching tubules and clear cell ribbons are reported to be the characteristic morphologic patterns [4]. The tubules, acini, ribbons, or papillae are lined with single layer of tumor cells. The tumor cells usually show moderate clear cytoplasm with low nuclear grade. The nuclei were oriented away from the basement membranes and toward the apical surfaces.

The tumors show a characteristic immunophenotype. The tumor cells are diffusely positive for CK7 and negative for alpha-methylacyl-CoA racemase (AMACR); the majority of the tumors are also negative for CD10 [4]. The stain for carbonic anhydrase IX (CA9) is variable.

 Genetically, the tumor does not show deletion of chromosome 3P. Aydin et al. showed that only 1 out of 10 tumors had low copy number gains of chromosomes 7 and 17 [4]. In addition, two tumors whose entire VHL gene sequences and promoter regions were analyzed showed no alterations [4].

CCPRCC usually presents with low pathologic stage. In 82 of reported cases, 80 were categorized as pathologic stage 1 and the remaining 2 tumors as pathologic stage 2 tumors [4, 5, 7–10]. The current case is pathologic stage 1. CCPRCC appears to be clinically indolent. Follow-up in 40 cases with a mean follow-up period of 28 months showed no evidence of disease after treatment [4]. In our case the patient is alive 21 months after the surgery.

The main differential diagnoses include clear cell renal cell carcinoma (CCRCC) and papillary renal cell carcinoma (PRCC). The primary morphological clue to CCPRCC is the recognition of papillary architecture. The tubules and cysts lined with clear cells may raise the suspicion for conventional CCRCC. However, CCPRCC lacks the delicate sinusoidal vascular networks seen in the CCRCC, while CCRCC does not have the branched tubules and complex clear cell ribbons seen in the former. In a difficult case, immunohistochemical stains can help to confirm the diagnosis. CCRCC is usually positive for RCC, CA9, and CD10 and negative for CK7 while CCPRCC is diffusely positive for CK7, commonly negative for CD10, and variably positive for CA 9. In the current case, we also stained the tumor for RCC; it was negative. Genetically, while CCRCC shows a deletion of chromosome 3p [11] and mutation in VHL gene in majority of sporadic cases [12], CCPRCC does not have such changes.

PRCC may show clear cell changes, which may be confused with CCPRCC. In addition to the characteristic morphological features, CCPRCC has unique immunohistochemical and genetic profiles. CCPRCC is diffusely positive for CK7, negative for AMACR, and also mostly negative for CD10 and variably positive for CA9, while PRCC is positive for CK7, CD10, and AMACR, usually negative for CA9. Gains of chromosome 7 and 17 are commonly seen in PRCC while such chromosomal changes were only seen in 1 out of 10 tested cases in CCRRCC [4].

Besides CCRCC and PRCC, several other renal tumors may enter in the differential diagnosis including multilocular cystic renal cell carcinoma (MLCRCC) and mixed epithelial and stromal tumor (MEST). MLCRCC is a variant of CCRCC with multilocular cystic growth pattern. The cysts are lined by clear cells with low nuclear grade [13, 14]. Genetically, MLCRCC also has chromosome 3p alteration [13]. Although CCPRCC can have abundant smooth muscle stroma, it does not, however, have the overall characteristic morphology of MEST.

Solid organ transplant recipients have higher risk for cancer development, which contributes to a far worse outcome than in the general population [1]. Renal transplant recipients have a 2–4-fold increase in overall cancer development [2, 3]. For kidney malignancy there is approximately a 15-fold increase [15]. The histologic types are approximately 2/3 conventional clear cell renal cell carcinoma and 1/3 of papillary renal cell carcinoma [16]. No clear cell papillary renal cell carcinoma has been previously reported in a renal transplant recipient. Renal cell carcinoma has been shown to be more aggressive tumors in renal transplant recipients compared to the general and dialysis population [17, 18], which may be partially attributed to the immunosuppression. The current case is the first case of CCPRCC reported in a kidney transplant recipient. The patient did not have tumor recurrence 21 months after surgery; however, the clinical behavior of this tumor in the kidney transplant recipients needs to be further evaluated with more cases and longer follow-up periods.
